# A cross-sectional analysis of yoga experience on variables associated with psychological well-being

**DOI:** 10.3389/fpsyg.2022.999130

**Published:** 2023-01-18

**Authors:** Tracie D. Parkinson, Stephen D. Smith

**Affiliations:** ^1^Department of Psychology, University of Manitoba, Winnipeg, MB, Canada; ^2^Department of Psychology, University of Winnipeg, Winnipeg, MB, Canada

**Keywords:** yoga, emotional regulation, mindfulness, self-compassion, interoception, spiritual intelligence

## Abstract

**Introduction:**

Previous research has identified numerous physical, psychological, and spiritual benefits associated with the practice of yoga. Indeed, yoga has been linked with improved quality of life, reduced stress, and numerous markers of psychological well-being. In the current research, a cross-sectional design was used to examine whether the psychological benefits associated with yoga only apply to long-term practitioners or whether more “casual,” intermittent yoga experience could produce positive outcomes.

**Methods:**

An American population of long-term practitioners (*n* = 129), intermittent practitioners (*n* = 161), and non-practitioners (*n* = 164) completed online self-report measures of emotional regulation, trait mindfulness, self-compassion, interoceptive awareness, and spiritual intelligence variables.

**Results:**

The results indicated that long-term (LT) practitioners scored higher than intermittent experience (IE) practitioners on measures of mindfulness (*M_LT_* = 137.3; *M_IE_* = 127.6), interoceptive awareness (*M_LT_* = 3.4; *M_IE_* = 3.1), self-compassion (*M_LT_* = 3.4; *M_IE_* = 3.1), and spiritual intelligence (*M_LT_* = 63.5; *M_IE_* = 55.5; all *p*-value < 0.05). Intermittent practitioners scored higher than no-experience (NE) group on interoceptive awareness (*M_IE_* = 3.1; *M_NE_* = 2.7) and spiritual intelligence (*M_IE_* = 55.5; *M_NE_* = 46.6; both *p*-value < 0.05). Contrary to our hypotheses, yoga experience had no effect on depression, anxiety, or stress levels. Separate mediation analyses demonstrated that interoceptive awareness, spiritual intelligence, mindfulness, and self-compassion each mediated the relationship between yoga experience and emotion dysregulation. Furthermore, emotion dysregulation mediated the relationship between yoga experience and depression, anxiety, and stress.

**Discussion:**

Taken together, the results of this study suggest that long-term practitioners experience more benefits compared to intermittent and non-practitioners, and that the mechanisms underlying these benefits are multi-faceted.

## 1. Introduction

Yoga originated in India over 5,000 years ago and has evolved into a popular activity that is practiced worldwide for a variety of physical, psychological, and spiritual purposes (Feuerstein, 2003; Pandurangi et al., 2017). Although yoga was originally performed in order to achieve a state of self-realization representing one’s greatest potential (Feuerstein, 2003), the primary focus of present-day yoga is on the numerous physical and emotional benefits that result from its practice (see Büssing et al., 2012, for a review). For example, yoga has been associated with improvements in self-reported quality of life (Birdee et al., 2017), well-being (Cartwright et al., 2020), and sleep quality (Sloan and Kanchibhotla, 2021). It has also been linked with more objective measures of physical health such as a lower waist/hip ratio and body mass index (see Lauche et al., 2016, for a review). Other research has demonstrated that yoga may enhance psychological well-being; yoga practitioners frequently score lower than non-practitioners on measures of perceived stress (Cartwright et al., 2020) and depression (Forseth et al., 2021; Sahni et al., 2021). However, although a rapidly expanding body of research has consistently highlighted the numerous potential benefits of yoga, few studies have included length of yoga practice as a variable of interest. As a result, it is unclear whether the benefits associated with yoga appear with intermittent practice (as would occur for “casual” practitioners) or if longer-term practice is required. The goal of the current research is to examine whether length of yoga practice is related to multiple measures of psychological health. For the purpose of this study, yoga is defined as a practice involving any combination of the following components or “limbs”: physical postures (Sanskrit: *asanas*), breathing techniques (*pranayama*), meditation, and/or ethical living toward oneself and others. These limbs may be practiced independently or in a classroom format with a certified instructor. In order to improve clarity, the term “yoga practice” is used to contrast individuals who performed yoga and those who have not; “yoga experience” refers to the length of time—typically months or years—that an individual has performed yoga.

A key component of yoga’s long-term psychological benefits appears to be its positive effects on emotional regulation (Gothe et al., 2019). Evidence from neuroimaging studies points to the fact that long-term yoga practice can have neuroplastic effects in areas related to emotional experiences and their regulation (van Aalst et al., 2020). For example, researchers have reported that yoga experience is associated with greater gray matter volume in multiple cortical regions—including the prefrontal cortex—as well as the cerebellum (Froeliger et al., 2012; Villemure et al., 2015; Afonso et al., 2017). This increased cortical volume is similar to that observed in previous studies of mindfulness meditation (Lazar et al., 2005; Tang et al., 2007) and suggests that long-term yoga practice may enhance executive control processes (Gothe et al., 2019). These yoga-related changes in the prefrontal cortex are particularly beneficial to older practitioners; changes in cortical thickness were negatively correlated with scores on the Cognitive Failures Questionnaire (Froeliger et al., 2012). Another volumetric brain-imaging study found that the volume of the right amygdala and left hippocampus were *smaller* in yoga practitioners (Gotnik et al., 2018). The fact that yoga decreases the volume of limbic structures related to emotional responses while also increasing the volume of brain areas involved with emotional regulation may explain the superior emotional regulation abilities reported in previous studies of yoga practitioners (e.g., Gothe et al., 2019).

Although both self-report and neuroimaging studies demonstrate the benefits of long-term yoga practice, there are still many questions that remain unanswered. For example, while yoga is associated with improvements in a number of negative emotional states, less is known about the interoceptive mechanisms that might mediate these effects. Additionally, it is currently unclear whether the benefits of yoga found in long-term practitioners would also be detected in individuals who take yoga classes in a less consistent manner (e.g., people who take yoga classes for some parts of the year but then have periods of time without practicing yoga). Addressing the first question would provide new insights into the factors underlying yoga’s benefits. Examining the second question would clarify whether the positive outcomes associated with yoga only apply to dedicated practitioners—or could be relevant to the more casual practitioner. It is important to note that we do not define dedicated practitioners as “experts” in this study. This study also aims to acknowledge traditional Eastern philosophy suggesting that humility and a beginner’s mind (*shoshin*) are important in a yoga practice in order to obtain benefits, particularly those of a spiritual nature, as a practitioner.

The current study used a cross-sectional design to investigate whether different measures of emotional well-being and three potential mediating mechanisms differ between individuals with no yoga experience, those with intermittent experience, and long-term yoga practitioners. Emotional well-being was quantified by using questionnaires that measured depression, anxiety, stress, and emotion dysregulation (Lovibond and Lovibond, 1995; Gratz and Roemer, 2004). To assess potential mechanisms of these changes, participants also completed self-report measures associated with mindfulness (Baer et al., 2006), interoceptive awareness (Mehling et al., 2012), and self-compassion (Neff, 2003). These specific variables were included because they are relevant to key components of yoga: physical postures (Sanskrit: *asanas*), breath regulation (*pranayama*), and meditation. Specifically, mindfulness and self-compassion relate to the meditative component of yoga whereas interoceptive awareness is related to the postural, breath regulation, and, sometimes, to the meditative elements of yoga. A measure of spiritual intelligence (King, 2008) was also included for exploratory purposes because many individuals report a spiritual motivation for practicing yoga (Park et al., 2016).

Our initial analyses examined whether there were differences between long-term practitioners, intermittent practitioners, and yoga on measures of psychological well-being. These analyses allowed us to compare our results with previous research; they also demonstrated that there were group differences that could be investigated with more sophisticated statistical analyses. Several mediation analyses were then performed in order to determine how *the relationships between these factors* vary as a function of yoga experience. Separate analyses were performed to test the hypotheses that mindfulness, self-compassion, and interoceptive awareness would each act as mediators underlying yoga’s beneficial effects on emotional dysregulation. We also predicted that mindfulness and self-compassion would act concurrently as mediators in the relationship between yoga experience and emotional regulation due to the top-down nature of these processes. Our final analyses examined whether emotional dysregulation could act as both an outcome variable (as noted above) *and* as a mediator of other effects. Specifically, we hypothesized that emotional dysregulation would mediate the relationship between yoga experience and depression, anxiety, and stress.

## 2. Materials and methods

### 2.1. Participants

The sample size for this study was determined using G*Power for effect size = 0.15, power = 0.80, and α = 0.05 for three groups. An estimate of 432 was provided, although more data were collected in the event of inaccurate reporting. A total of 492 participants completed the current study. Participants were recruited through the Qualtrics Online Market Research Panel (Qualtrics, Inc., Provo, UT) between April and August 2021. Participants were at least 18 years of age and fell into one of three groups: individuals with no experience practicing yoga in their lifetime, individuals who practiced yoga intermittently, or individuals who engaged in a dedicated yoga practice. Participants were provided with these options in multiple-choice format. Intermittent yoga practice was defined as phases of consistent practice (at least 45 min, once each week for several months or years) mixed with phases (months or years) of little to no practice. The intermittent yoga group was included in this study because many individuals have *some* yoga experience, but tend to take occasional classes rather than incorporating yoga regularly into their lifestyle. Long-term practice was defined as individuals with a minimum of 3 years or more of regular yoga practice (at least 45 min, once each week).

This study received ethics approval from the Human Research Ethics Board at the University of Manitoba (Fort Garry Campus). All participants provided informed consent prior to participating in this experiment.

### 2.2. Procedure

After providing informed consent, participants provided demographic information and completed six questionnaires. The questionnaires were presented in random order. These included the Depression, Anxiety, and Stress Scale (DASS-42; Lovibond and Lovibond, 1995), Difficulties in Emotion Regulation Scale (DERS; Gratz and Roemer, 2004), Five-Facet Mindfulness Questionnaire (FFMQ; Baer et al., 2006), Multidimensional Assessment of Interoceptive Awareness (MAIA; Mehling et al., 2012), Self-Compassion Scale (SCS; Neff, 2003), and The Spiritual Intelligence Self-Report Inventory (SISRI-24; King, 2008). In order to detect the possibility of fatigue, additional statements were added to each questionnaire at random intervals. An example of these statements includes, “Please select 3” or, “Please select 1.” No participants responded incorrectly to these statements.

Specific information was also collected from the intermittent and long-term yoga groups on their yoga practice. This information included which limb(s) participants practiced, with the possibility of selecting *asanas*, *pranayama*, meditation, ethical living (toward oneself and others), or any combination of these responses. Participants also indicated which schools of yoga they have practiced, with the opportunity to select any combination of the following: *Ashtanga*, Bikram, *Hatha*, Hot Yoga (non-Bikram style), Integral, Iyengar, *Kripalu*, *Kundalini*, Power, Restorative, *Vinyasa*, Yin, Other, or Unsure. Lastly, information was collected on the format in which participants practiced. Participants were selected if they engaged in formal practices (classes led by a certified Yoga instructor for an individual or a group in-person or live online), informal practices (non-video home practice, YouTube videos, or self-guided through books), or both.

### 2.3. Questionnaires

#### 2.3.1. Depression, anxiety, and stress scale

The Depression, anxiety, and stress scale (DASS-42) is a 42-item measure of state depression, anxiety, and stress (Lovibond and Lovibond, 1995). Participants indicate on a 4-point Likert scale the degree to which each statement applies over the past week, from 0 (*Did not apply to me at all*) to 3 (*Applied to me very much, or most of the time*). The 42 items consist of 14 statements each corresponding to a measure of depression (MacDonald’s ω = 0.97), anxiety (ω = 0.95), or stress (ω = 0.96).

#### 2.3.2. Difficulties in emotional regulation scale

The Difficulties in emotional regulation scale (DERS) is a 36-item self-report measure of emotional regulation (Gratz and Roemer, 2004). Participants indicate on a 5-point Likert scale (*1 = Almost Never (0%–10%); 2 = Sometimes (11%–35%); 3 = About Half the Time (36%–65%); 4 = Most of the Time (66%–90%); 5 = Almost Always (91%–100%)*) the degree to which each of the 36 statements apply to them. Higher scores indicate greater difficulties in emotional regulation. The DERS produces one overall score which encompasses all items as well as scores for six sub-scales. The six constructs of the DERS include, with abbreviations in parentheses, Nonacceptance of Emotional Responses (*Non-Accept*; ω = 0.91), Difficulties Engaging in Goal-Directed Behavior (*Goals*; ω = 0.88), Impulse Control Difficulties (*Impulse*; ω = 0.90), Lack of Emotional Awareness (*Awareness*; ω = 0.85), Limited Access to Emotion Regulation Strategies (*Strategies*; ω = 0.92), and Lack of Emotional Clarity (*Clarity*; ω = 0.79; Gratz and Roemer, 2004). Only the total scores were used in the present investigation.

#### 2.3.3. Five-facet mindfulness questionnaire

The Five facet mindfulness questionnaire (FFMQ) measures the tendency to exhibit mindfulness as a personality trait (also called dispositional or trait mindfulness; Baer et al., 2006). The FFMQ consists of 39 statements; participants rate each statement on a 5-point Likert scale, from *1 = never or very rarely true* to *5 = very often or always true*. The FFMQ produces an overall score of trait mindfulness and scores for each of the five facets; higher scores delineate greater trait mindfulness. The five facets consist of *Observing* (paying attention to both internal and external sensations; ω = 0.86), *Describing* (the ability to describe inner experiences; ω = 0.84), *Acting with Awareness* (mindful engagement in activities; abbreviated as *Acting*; ω = 0.90), *Non-Judgment of Inner Experiences* (treating one’s internal experiences with openness and curiosity rather than with negative evaluations; abbreviated as *Non-Judging*; ω = 0.91), and *Non-Reacting to Inner Experience* (the ability to remain objective, separate, and calm while one experiences thoughts and emotions; abbreviated as *Non-Reacting*; ω = 0.84; Baer et al., 2006). For the current study, only the total mindfulness scores were utilized.

#### 2.3.4. Multidimensional assessment of interoceptive awareness

The Multidimensional assessment of interoceptive awareness (MAIA) consists of 32 items in an eight-factor model where individuals rank on a 6-point Likert scale the degree to which each statement applies (*0 = Never; 5 = Always*; Mehling et al., 2012). The MAIA is calculated using a total score, corresponding to greater body awareness, and eight sub-scale scores, corresponding to each construct. The eight-subscales of the MAIA consist of *Noticing* (being aware of one’s bodily sensations; ω = 0.83), *Not-Distracting* (not ignoring or avoiding uncomfortable sensations; ω = 0.63), *Not-Worrying* (not experiencing distress during unpleasant or painful sensations; ω = 0.64), *Attention Regulation* (paying attention to and controlling bodily sensations; ω = 0.91), *Emotional Awareness* (connecting bodily sensations with emotions; ω = 0.89), *Self-Regulation* (attending to body sensations in order to regulate distress; ω = 0.88), *Body Listening* (using information from the body to inform decisions; ω = 0.88), and *Trusting* (trusting the validity of one’s bodily sensations; ω = 0.89; Mehling et al., 2012). Only the total interoceptive awareness scores were used in the present investigation.

#### 2.3.5. Self-compassion scale

The Self-compassion scale (SCS) is a 26-item self-report measure developed by Neff (2003). Individuals indicate on a five-point Likert scale from *1 (almost never) to 5 (almost always)* the degree to which each statement applies. The SCS is comprised an overall score indicating a general factor of self-compassion as well as six sub-scales. These subscales include *Self-Kindness* (being understanding and supportive to oneself; ω = 0.87), *Self-Judgment* (a person’s tendency to judge themselves for their limitations; ω = 0.89), *Common Humanity* (the ability to recognize that personal difficulties or inadequacies are aspects of life experienced by everyone; ω = 0.82), *Isolation* (the tendency to feel alone or isolated following a mistake or when experiencing difficulties; ω = 0.86), *Mindfulness* (the ability to live in the present moment; ω = 0.84), and *Over-Identification* (the tendency to get “carried away” by negative elements of one’s life; ω = 0.85). Self-Judgment, Isolation, and Over-identification are all reversed scores. Greater scores on the SCS indicate greater self-compassion (Neff, 2003). For the current study, only the overall SCS score was used.

#### 2.3.6. Spiritual intelligence self-report inventory

The Spiritual intelligence self-report inventory (SISRI-24) is a 24-item questionnaire that provides a measure of spiritual intelligence (King, 2008). An overall score is calculated by adding all items in the questionnaire, following reverse scoring of one item. A higher score is indicative of greater spiritual intelligence. Four sub-scales (King and DeCicco, 2009) are included in this questionnaire, including *Critical Existential Thinking* (*CET*; the ability to reflect on existential topics such as meaning, purpose, and death; ω = 0.87), *Personal Meaning Production* (*PMP*; the capacity to generate a sense of meaning, control, and mastery of life; ω = 0.88), *Transcendental Awareness* (*TA*; perceiving transcendent aspects of the Self, others, and the world that contribute to a sense of interconnectedness; ω = 0.87), and *Conscious State Expression* (*CSE*; the capacity to freely travel to and from other states of consciousness; ω = 0.93). Higher scores on these sub-scales indicate greater capacity to engage in these aspects that contribute to spiritual intelligence (King, 2008). Only the total scores were used in the present investigation.

### 2.4. Data analysis

All analyses were performed using IBM SPSS Statistics 28.0 Software. One-way ANOVAs were performed for all analyses. Assumptions were tested for normality (Shapiro–Wilk test, *p* > 0.05), homogeneity of variances (Levene’s test, *p* > 0.05), and outliers (inspection of boxplots). Data demonstrating a combination of non-normal distributions and outliers were transformed. If outliers were present, data were left due to the strong likelihood that it represented genuinely unusual values. If variances were heterogeneous, results of a Welch ANOVA were reported instead. The Tukey–Kramer post-hoc test was used for most ANOVAs because it allows for unequal sample sizes. If the assumption of homogeneity of variances was violated (Levene’s test, *p* < 0.05), the Games–Howell *post-hoc* test was interpreted instead.

## 3. Results

### 3.1. Demographic variables

A total of 38 responses were removed due to incomplete or inaccurate reporting. Therefore, a total of 454 participants remained: (n_no experience_ = 164; n_intermittent_ = 161; n_long-term_ = 129). The demographic characteristics of these participants are included in Tables 1, 2. Information regarding gender was missing for up to 35% of the participants in a given subgroup and was not considered further. Information about the participants’ yoga experience is summarized in Table 3.

**Table 1:** Participants’ yoga, meditation, and praying experience [mean (M) and standard deviation (SD)].

	No experience		Intermittent		Long-term	
	M	SD	M	SD	M	SD
Age	44.5	8.7	46.9	17.4	48.0	16.0
Lifetime yoga experience (years)	N/A	N/A	7.0	8.7	11.9	9.9
Past year yoga experience (hours)	N/A	N/A	49.1	73.1	237.8	397.4
Lifetime meditation experience (years)	3.1	8.2	7.0	9.5	10.8	11.4
Past year meditation experience (hours)	42.9	247.9	57.1	80.2	145.6	285.7
Lifetime experience praying (years)	21.7	19.1	25.0	22.0	23.8	22.6

**Table 2:** Participant demographic information (percentage).

		No experience (%)	Intermittent (%)	Long-term (%)
Education	Some high school	7.3	2.5	0.8
	High school diploma	32.9	12.4	7.0
	Some college/university	24.4	28.6	18.6
	Diploma or degree	29.3	41.6	47.4
	Graduate degree	6.1	13.7	26.4
	Other	0.0	1.2	0.0
Ethnicity	White	82.9	83.2	85.3
	Black	7.3	7.5	7.0
	Latin American	3.0	3.7	2.3
	South Asian	0.0	0.6	2.3
	Filipino	1.2	0.0	0.8
	Indigenous	0.0	0.6	1.6
	Chinese	0.0	0.6	0.8
	Korean	0.6	0.6	0.0
	Japanese	0.6	0.6	0.0
	Arab/West Indian	0.6	0.0	0.0
	Southeast Asian	0.6	0.0	0.0
	Other	3.0	2.5	0.0
Religious/spiritual affiliation	Agnostic/atheist/secular	7.3	11.8	6.2
	Buddhism	0.6	1.2	1.6
	Christian	61.0	60.2	59.7
	Hinduism	0.0	0.6	3.1
	Indigenous	0.0	0.0	0.0
	Judaism	0.6	0.6	3.1
	Muslim	0.6	0.0	3.9
	Other	7.9	7.5	7.0
	None Indicated	22.0	18.0	15.5

**Table 3:** Participant yoga experience (percentage of participants practicing each limb, school, and format).

		Intermittent (%)	Long-term (%)
Limb^*^	Asanas	47.8	62.8
	Pranayama	55.3	63.6
	Meditation	70.8	71.3
	Ethical living	32.9	39.5
School of yoga^*^	Ashtanga	5.0	16.3
	Bikram	7.5	16.3
	Hatha	9.3	20.2
	Hot (non-Bikram)	6.8	14.0
	Integral	3.1	14.7
	Iyengar	1.9	11.6
	Kripalu	0.0	7.8
	Kundalini	4.3	14.7
	Power	9.9	26.4
	Restorative	17.4	31.0
	Vinyasa	9.9	33.3
	Yin	4.3	18.6
	Other	5.6	6.2
	Unsure	56.5	14.0
Format of practice	Formal	8.7	8.5
	Informal	63.4	28.7
	Both	28.0	62.8

* Participants were provided the opportunity to select more than one response.

A One-Way ANOVA showed that there was no statistically significant difference among groups with regard to age [*F*(2, 451) = 2.43, *p* = 0.09] or number of years praying [*F*(2, 451) = 1.03; *p* = 0.36]. Meditation experience differed among the groups in terms of hours in the past year [*F*(2, 451) = 9.00; *p* < 0.001] and years in their lifetime [*F*(2, 451) = 22.24; *p* < 0.001]. Post-hoc analysis using the Tukey–Kramer test demonstrated that the long-term group reported more years (*p* < 0.001) and hours in the past year meditating (*p* = 0.004) compared to the no-experience group. Long-term practitioners also reported more years meditating (*p* = 0.008) and hours meditating in the past year (*p* = 0.002) compared to the intermittent group. The intermittent group demonstrated significantly more meditation experience than the no-experience group in years (*p* < 0.001); however, hours meditating in the past year did not differ between the two groups (*p* = 0.77). As expected, using Independent Samples *t*-test, yoga experience differed, with the long-term group reporting more hours in the past year [*t*(135) = −5.32; *p* < 0.001] and years in their lifetime [*t*(188) = −4.55; *p* < 0.001] compared to the intermittent group. Because age and years of prayer were not significantly different among groups, no covariates were used in all subsequent analyses. Meditation history was not used as a covariate due to meditation being a significant part of most yoga practices (Feuerstein, 2003, 2008).

### 3.2. Total score profiles

A summary of the scores of all scales is included in Table 4 below. One-way ANOVAs were conducted for the total score of each measure to identify differences among the three groups. The Tukey–Kramer test was used to identify differences among groups if the ANOVA was significant. A correction was used to account for multiple comparisons. Because three groups were compared, an adjusted *p*-value of 0.05/3 was used as the cut-off.

**Table 4:** Summary of scores [mean (M) and median].

	No experience		Intermittent		Long-term	
	M (SE)	Median	M (SE)	Median	M (SE)	Median
Depression (DASS-42)	12.2 (0.9)	9.0	12.0 (1.0)	7.0	10.6 (1.0)	6.0
Anxiety (DASS-42)	9.7 (0.7)	6.0	10.8 (0.8)	8.0	10.3 (0.9)	6.0
Stress (DASS-42)	12.6 (0.8)	10.5	13.9 (0.9)	11.0	12.1 (1.0)	8.0
DERS	91.2 (2.1)	91.0	85.2 (2.2)	82.0	78.8 (2.5)	71.0
FFMQ	121.2 (1.5)	118.0	127.6 (1.7)	125.0	137.3 (2.1)	137.0
SCS	3.0 (0.06)	3.0	3.1 (0.06)	3.1	3.4	3.2
MAIA	2.7 (0.06)	2.8	3.1 (0.7)	3.0	3.4 (0.06)	3.4
SISRI-24	46.6 (1.6)	46.0	55.5 (1.3)	56.0	63.5 (1.7)	65.0

The results for the DASS-42 did not indicate any differences among groups. Depression, Anxiety, and Stress scores from the DASS-42 were not significant (all *F-*values < 1). We analyzed the proportion of DASS-42 scores that fell in a clinically significant range (moderate, severe, or extremely severe; Table 5). There was a trend for the long-term depression, anxiety, and stress scores to have fewer proportions of clinically significant scores compared to the intermittent and no-experience groups. The intermittent group had a trend for a lower proportion of scores in the clinically significant range for depression. This trend was not seen when comparing the intermittent and no-experience groups for anxiety and stress. These scores were compared using a Chi-squared test. No scores were significant (χ^2^ < 1.20; *p* < 0.05), indicating that there were no differences in the proportions of scores in the clinically significant range among the groups.

**Table 5:** DASS-42 results and clinical interpretation.

	Depression			Anxiety			Stress		
	M	SD	% CS	M	SD	%CS	M	SD	%CS
No experience	12.2	11.8	40.2	9.7	9.3	43.3	12.6	9.9	29.3
Intermittent	12.0	12.4	36.6	10.8	10.1	43.5	13.9	11.0	29.2
Long-term	10.6	11.7	34.1	10.3	10.5	41.9	12.1	11.5	26.4

%CS, percentage of scores in the clinically significant range (moderate, severe, or extremely severe). Depression scores in this range are from 14 to 42. Anxiety scores in this range are 10–42. Stress scores in this range are 19–42.

**Figure 1:**
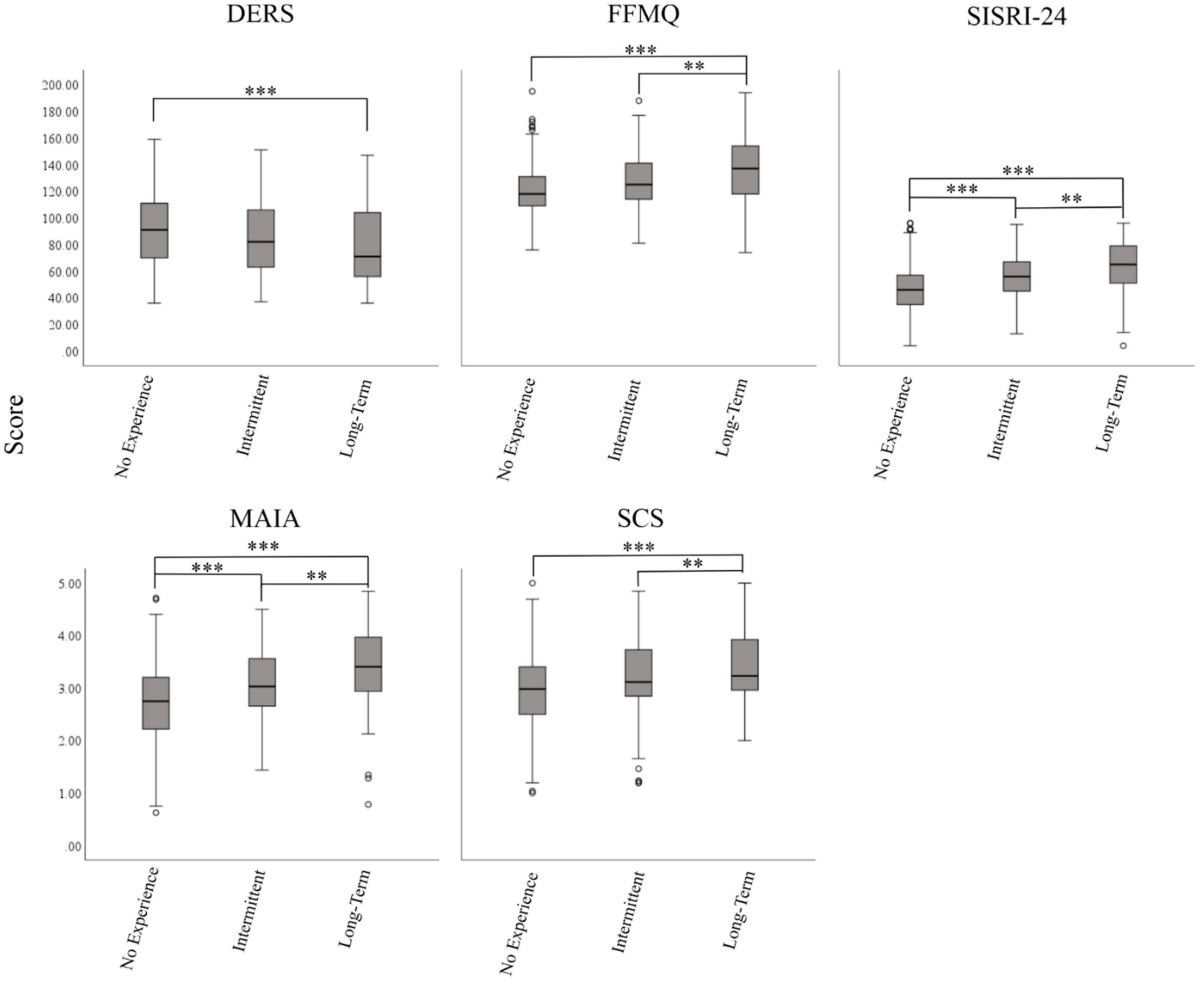
ANOVA summary. Margin bars represent ±5% of the mean score. Significance is indicated with ^**^p < 0.01 and ^***^p < 0.001.

The one-way ANOVA results were significant for the remaining five questionnaires; however, planned comparisons indicated that not all groups differed from one another. For the DERS, the overall ANOVA was significant: [*F*(2,451) = 7.24, *p* < 0.001, ƞ_p_^2^ = 0.31]. As expected, the long-term experience group had lower scores compared to the no-experience group on this measure (*p* < 0.001). However, there were no significant differences on the DERS between the long-term and intermittent groups or between the intermittent and no-experience groups (see Figure 1).

**Figure 2:**
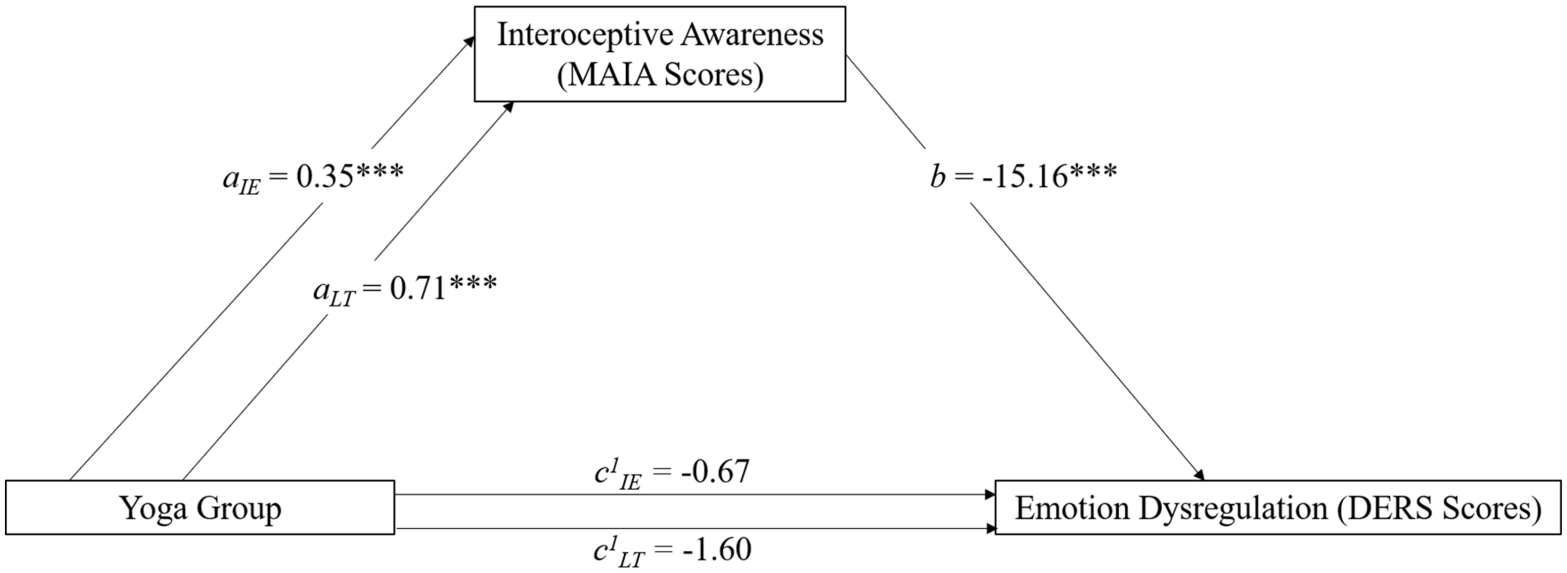
Mediation model featuring yoga practice (predictor), emotion dysregulation (outcome), and interoceptive awareness (mediator). Variables with subscript IE represent a comparison between the no experience and intermittent experience (IE) group. Subscript LT represents a comparison between the no experience and the long-term (LT) group. This model demonstrates that the relationship between intermittent and long-term yoga practice and emotion dysregulation is mediated by interoceptive awareness. *** denotes significance with *p* < 0.001. No asterisk represents a non-significant finding.

The one-way ANOVAs for the FFMQ [*F*(2,451) = 20.88, *p* < 0.001, ƞ_p_^2^ = 0.085] and the SCS [*F*(2,451) = 12.95, *p* < 0.001, ƞ_p_^2^ = 0.054] were both significant (see Figure 2). For both of these measures, the long-term group had higher scores than the no experience (*p* < 0.001) and the intermittent experience (*p* < 0.006) groups. However, no significant difference was found between the intermittent and no-experience groups on either the FFMQ or the SCS, although there was a trend for significance for the FFMQ (*p* = 0.019).

**Figure 3:**
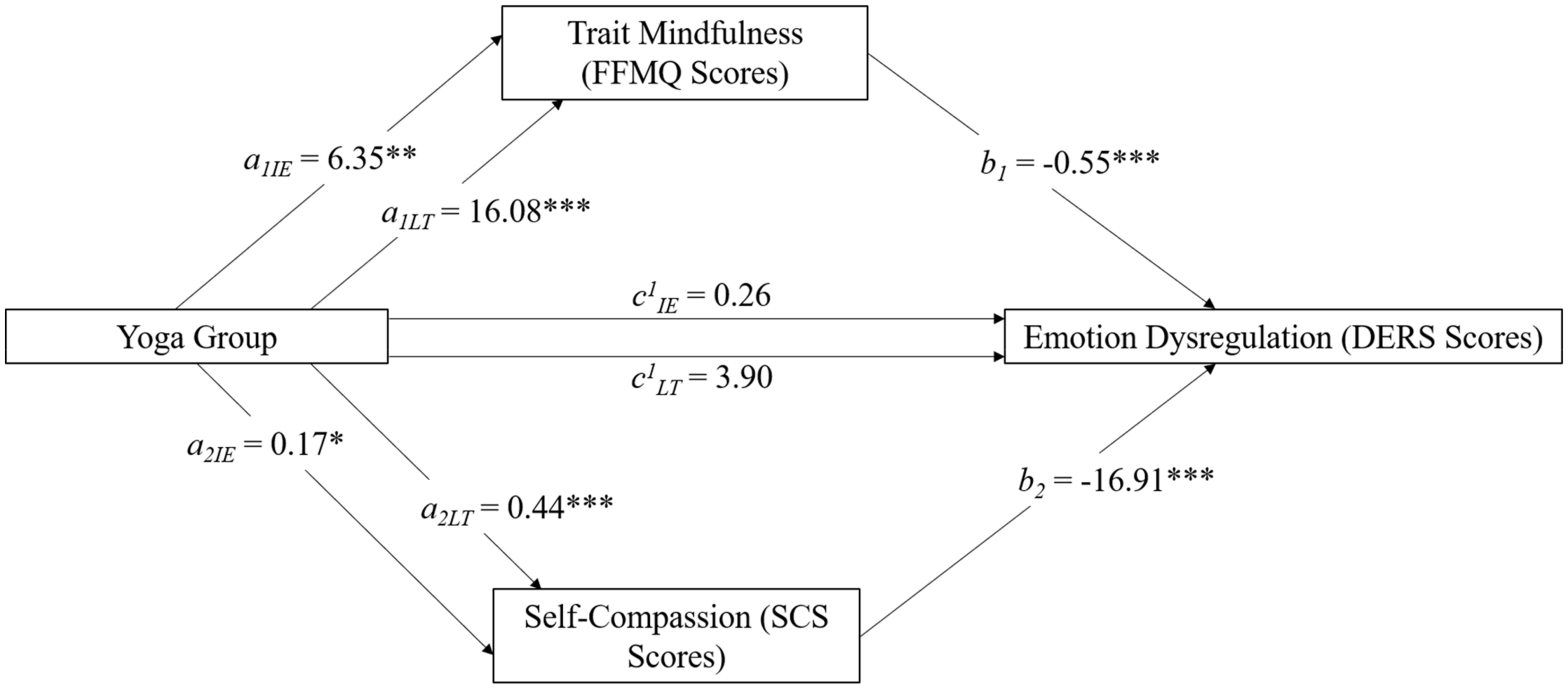
Mediation model featuring yoga practice (predictor), emotion dysregulation (outcome), and two mediators (self-compassion and trait mindfulness). Variables with subscript IE represent a comparison between the no experience and intermittent experience (IE) group. Subscript LT represents a comparison between the no experience and the long-term (LT) group. This model demonstrates that the relationship between intermittent and long-term yoga practice and emotion dysregulation is mediated by trait mindfulness and self-compassion. Significance is represented as * (*p* < 0.05), ** (*p* < 0.01), or *** (*p* < 0.001). No asterisk represents a non-significant finding.

In contrast, all groups differed from one another on the MAIA and the SISRI. The one-way ANOVAs for the MAIA [*F*(2,451) = 34.09, *p* < 0.001, ƞ_p_^2^ = 0.13] and the SISRI [*F*(2,451) = 28.95, *p* < 0.001, ƞ_p_^2^ = 0.11] were significant (see Figure 3). For both of these measures, the long-term group had higher scores than the no-experience group (*p* < 0.001) and the intermittent experience group (*p* < 0.006), and the intermittent group had greater scores compared to the no-experience group (*p* < 0.001).

### 3.3. Mediation analyses

Mediation analyses were performed using PROCESS v3.5 in SPSS. The long-term and intermittent groups were compared to the no-experience group only. These comparisons were used to determine whether the mechanisms of yoga’s benefits are the same for both long-term and intermittent practitioners.

More broadly, mediation was used to investigate which aspects of a yoga practice (trait mindfulness, interoception, and self-compassion) contribute to health benefits (reducing depression, anxiety, stress, and/or emotion dysregulation). Five mediation models were tested, each representing separate hypotheses. Yoga group represented the predictor variable for each model, whereas the mediators and outcomes varied. Yoga group was dummy coded in PROCESS to account for the categorical nature of this variable. Comparisons were between the no-experience group and the intermittent group, and between the no experience and the long-term group. These comparisons are delineated with separate paths and coefficients (IE representing the intermittent experience group and LT representing the long-term practitioner group).

**Figure 4:**
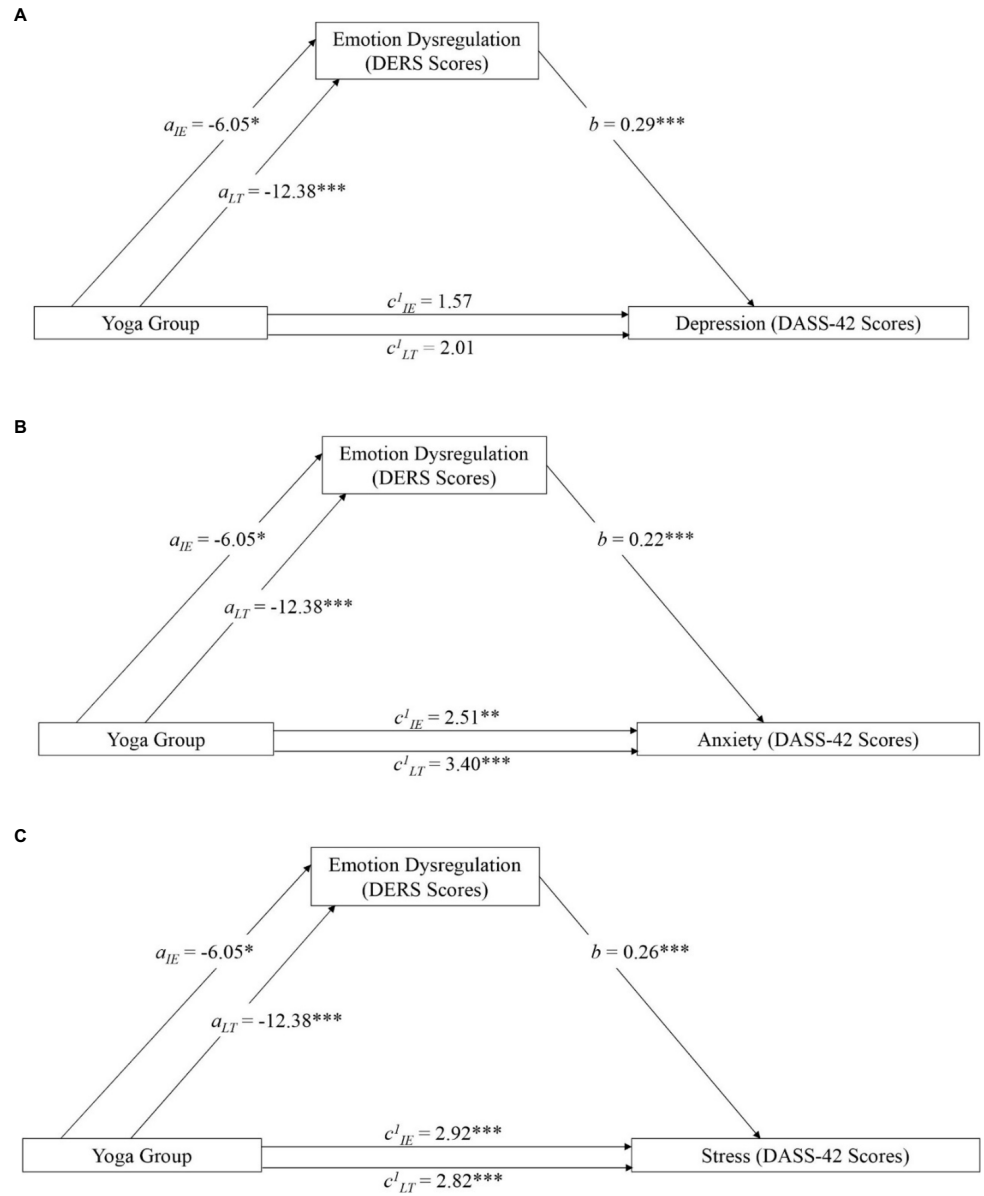
Mediation model featuring yoga practice (predictor), emotion dysregulation (mediator), and depression **(A)**, anxiety **(B)**, and stress (**C**; outcomes). Variables with subscript IE represent a comparison between the no experience and intermittent experience (IE) group. Subscript LT represents a comparison between the no experience and the long-term (LT) group. Significance is represented with * (*p* < 0.05), ** (*p* < 0.01), or *** (*p* < 0.001).

The first model tested our hypothesis that interoceptive awareness (MAIA total score) would mediate the relationship between yoga practice (group) and emotion dysregulation (DERS total score). That is, the relationship between yoga practice (intermittent and long-term) and emotion dysregulation would show lesser or no significance when introducing interoceptive awareness as a variable that explains this relationship. Model 4 in PROCESS was used. Models for both the intermittent (Indirect effect = −0.19; SE = 0.047; 95% CI [−0.29, −0.11]) and long-term (Indirect effect = −0.38; SE = 0.064; 95% CI [−0.52, −0.26]) groups were significant (Figure 4).

**Figure 5:**
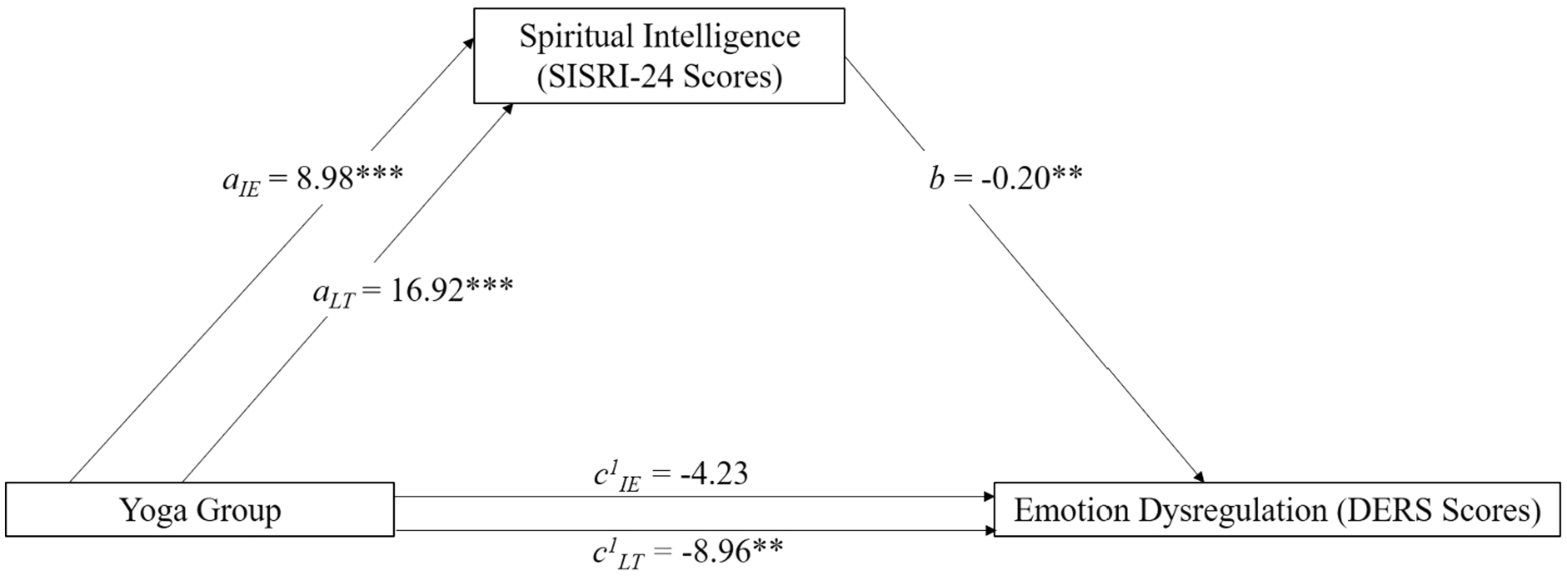
Mediation model featuring yoga practice (predictor), emotion dysregulation (outcome), and spiritual intelligence (mediator). Variables with subscript IE represent a comparison between the no experience and intermittent experience (IE) group. Subscript LT represents a comparison between the no experience and the long-term (LT) group. Significance is represented with ** (*p* < 0.01) or *** (*p* < 0.001). No asterisk represents a non-significant finding.

Our second hypothesis was that self-compassion (SCS total score) and trait mindfulness (FFMQ total score) would *concurrently* mediate the relationship between yoga experience (group) and emotion dysregulation (DERS). Model 4 in PROCESS was used. Comparisons between the no experience and intermittent group (FFMQ Indirect effect = −3.49; SE = 1.28; 95% CI[−6.07, −1.00]; SCS Indirect effect = −0.12; SE = 0.046; 95% CI [−0.36, −0.22]) and the no experience with the long-term group (FFMQ Indirect effect = −8.83; SE = 1.60; 95% CI [−12.09, −5.83]; SCS Indirect effect = −0.31; SE = 0.06; 95% CI [−0.43, −0.21]) were significant (Figure 5).

The remaining three hypotheses were that emotion dysregulation (DERS total score) would mediate the relationship between yoga experience and depression, anxiety, and stress. All models were significant (Figure 4). The model for depression was significant for both the intermittent (Indirect effect = −1.74; SE = 0.87; 95% CI = [−3.41, −0.37]) and long-term (Indirect effect = −3.57; SE = 0.97; 95% CI = [−5.48, −1.64]) groups. The model for anxiety was significant for both the intermittent (Indirect effect = −1.36; SE = 0.68; 95% CI = [−2.68, −0.17]) and long-term (Indirect effect = −2.78; SE = 0.75; 95% CI = [−4.26, −1.30]) groups. The model for stress was significant for the intermittent (Indirect effect = −1.59; SE = 0.79; 95% CI = [−3.14, −0.48]) and long-term (Indirect effect = −3.24; SE = 0.87; 95% CI = [−5.02, −1.59]) groups.

Exploratory mediation analyses were also conducted. The initial analyses used model 4 in PROCESS. First, we tested the hypothesis that interoceptive awareness (MAIA) would mediate the relationship between yoga experience and spiritual intelligence (SISRI). This relationship was significant for both the intermittent (Indirect effect = 0.22; SE = 0.052; 95% CI [0.12, 0.32]) and long-term (Indirect effect = 0.42; SE = 0.065; 95% CI [0.29, 0.55]) groups. This initial analysis was a precursor to the following analyses. Because interoceptive awareness mediated the relationship between yoga experience and both spiritual intelligence and emotion dysregulation, we conducted a second analysis to see if spiritual intelligence would mediate the relationship between yoga experience and emotion dysregulation. This mediation model was also significant for the intermittent (Indirect effect = −1.82; SE = 0.77; 95% CI = [−3.48, −0.45]) and long-term (Indirect effect = −3.43; SE = 1.35; 95% CI [−6.31, −0.93]) groups (Figure 5). These analyses demonstrate that the pathways for yoga-producing benefits are the same for long-term and intermittent practitioners, with interoceptive awareness, mindfulness, self-compassion, and spiritual intelligence being important mediators for emotion dysregulation. A summary of the mediation analyses is provided in Table 6.

**Table 6:** Mediation analysis summary.

		Total effect	Direct effect	*p*-value	Indirect effect	95% CI
Analysis 1	Intermittent	−6.1	−0.7	0.05	−0.2	−0.3, −0.1
	Long-term	−12.4	−1.6	0.0002	−0.4	−0.5, −0.3
Analysis 2	Intermittent	−6.1	0.3	0.9	−3.5 (FFMQ) −2.8 (SCS)	−6.1, −1.2 (FFMQ) −12.2, −5.8 (SCS)
	Long-term	−12.4	3.9	0.05	−2.9 (FFMQ) −7.4 (SCS)	−5.7, −0.04 (FFMQ) −10.5, −4.5 (SCS)
Analysis 3	Intermittent	−0.2	1.6	0.1	−1.7	−3.4, −0.4
	Long-term	−1.6	2.0	0.06	−3.6	−5.5, −1.6
Analysis 4	Intermittent	1.2	2.5	0.004	−1.4	−2.7, −0.2
	Long-term	0.6	3.4	0.0003	−2.8	−4.3, −1.3
Analysis 5	Intermittent	1.3	2.9	0.001	−1.6	−3.1, −0.5
	Long-term	−0.4	2.8	0.003	3.2	−5.0, −1.6
Analysis 6	Intermittent	9.0	3.1	0.06	5.9	3.1, 8.7
	Long-term	16.9	5.2	0.005	11.7	8.2, 15.3
Analysis 7	Intermittent	−6.1	−4.2	0.2	−1.8	−3.5, −0.5
	Long-term	−12.4	−9.0	0.009	−3.4	−6.4, −1.0
Analysis 8	Intermittent	−6.1	−1.6	0.6	1.7	0.6, 3.1
	Long-term	−12.4	−3.1	0.3	3.3	1.4, 5.8
Analysis 9	Intermittent	−6.1	−1.6	0.6	−4.4	−6.7, −2.4
	Long-term	−12.4	−3.1	0.3	−8.3	−11.4, −5.6

Analysis 1 consisted of the MAIA (mediator) and DERS (outcome). Analysis 2 consisted of the SCS and FFMQ (concurrent mediators) and DERS (outcome). Analysis 3 consisted of the DERS (mediator) and DASS-42 Depression scale (outcome). Analysis 4 consisted of the DERS (mediator) and DASS-42 Anxiety scale (outcome). Analysis 5 consisted of the DERS (mediator) and DASS-42 Stress scale (outcome). Analysis 6 consisted of the MAIA (mediator) and SISRI-24 (outcome). Analysis 7 consisted of the SISRI-24 (mediator) and DERS (outcome). Analysis 8 consisted of the MAIA and SISRI-24 (sequential mediators) and DERS (outcome). Analysis 9 consisted of the SISRI-24 and MAIA (sequential mediators) and DERS (outcome).

## 4. Discussion

The current study demonstrates that long-term yoga practice is associated with a number of positive outcomes. Relative to non-practitioners and intermittent yoga practitioners, long-term practitioners showed higher levels of self-reported trait mindfulness, interoceptive awareness, self-compassion, emotional regulation abilities, and spiritual intelligence. The diversity of these benefits is noteworthy and suggests that yoga *may* enhance not only body awareness, but also some executive functions (e.g., mindful attention and emotional regulation). Importantly, many of these benefits were not limited to long-term practitioners of yoga—intermittent practitioners also showed higher levels of interoceptive awareness and spiritual intelligence compared to non-practitioners. These results suggest that even inconsistent yoga practice can lead to positive outcomes. It is important to note, however, that the design of the current study does not allow us to make claims about causality; the presence or absence of yoga was not manipulated in an experimental design. It is possible that our results indicate which cluster of personality traits increase the likelihood that someone will consistently practice yoga rather than reflecting the benefits of having practiced yoga. Therefore, for the subsequent discussion of these data, we will focus on traits *associated with* yoga experience. Any discussion of causality will be identified as being speculative.

A key result of the current study related to emotion dysregulation; long-term practitioners had lower scores on the DERS than the intermittent and non-practitioner groups. Our findings are consistent with previous research that reported improvements in emotional regulation following 2 weeks of yoga-based meditation (Patel et al., 2018), short-term yoga interventions for adolescents (Daly et al., 2015; McMahon et al., 2021), 6 weeks of yoga for incarcerated individuals (Willy-Gravley et al., 2021), and a minimum of 1 year of yoga practice (Kobylińska et al., 2018). Given that many of these earlier studies involved improvements arising from yoga training, it is likely that the group differences observed in the current research represent a benefit associated with long-term yoga practice. Importantly, the benefits to emotional regulation do not appear to be due to physical activity alone (Daly et al., 2015). One possible mechanism of emotional regulation through yoga practice is cognitive reappraisal (Menezes, 2015; Kobylińska et al., 2018). Menezes (2015) suggested that yoga teaches attention allocation and acceptance, antidotes to rumination, and impulsive reactivity that may contribute to cognitive reappraisal. Future studies could address this possibility by specifically examining cognitive reappraisal—along with other emotional regulation strategies (see McRae and Gross, 2020, for a review)—before and after a yoga training program.

Long-term practitioners also differed from the intermittent and non-practitioner groups on a measure of trait mindfulness. These data are consistent with earlier research showing that the number of months participants practiced yoga positively correlated with FFMQ scores (Snaith et al., 2018). Importantly, our data are also consistent with experimental studies in which participants were measured before and after yoga training. For example, Erkin and Aykar (2021) reported greater scores on the FFMQ in nursing students following 14 weeks of yoga practice. Another study reported improved mindful attention as measured by the Mindful Attention Awareness Scale (MAAS; Brown and Ryan, 2003) following 2 weeks of daily yoga-based meditation practice (Patel et al., 2018). Taken together, the current study, along with these previous investigations, suggests that yoga practice can enhance trait mindfulness.

The results of our measure of interoceptive awareness were particularly interesting given the key role that bodily awareness plays in most forms of yoga. In our study, long-term practitioners reported higher interoceptive awareness (as shown by scores on the MAIA) than the intermittent and non-practitioner groups. Intermittent practitioners also reported higher interoceptive awareness scores compared to non-practitioners, suggesting that even inconsistent yoga practice may improve awareness of internal bodily sensations. This beneficial effect of yoga is consistent with the results of previous studies using clinical populations. For example, the combination of breath work, mindfulness, and mindful movement contributed to greater MAIA scores in war veterans diagnosed with Post-Traumatic Stress Disorder (PTSD; Mehling et al., 2018). Future research could investigate the relationships between specific elements of yoga (e.g., breathing or postures) on specific subcomponents of interoceptive awareness. These studies could also examine whether yoga practice influences interoception on objective measures such as heartbeat detection rather than self-report measures such as the MAIA.

Similar to interoceptive awareness, self-compassion scores were higher in the long-term practitioner group, but also significantly higher in the intermittent group than in the non-practitioner participants. These results are consistent with other studies incorporating self-compassion as a construct, including a study of the effects of a 3-day residential yoga program on professional educators (Dyer et al., 2021), an examination of the effects of a 2-week yoga-based meditation program (Patel et al., 2018), a study of nursing students following 14 weeks of yoga practice (Erkin and Aykar, 2021), and an examination of regular yoga practitioners (Snaith et al., 2018). Together, these studies suggest that self-compassion scores may improve with either short-or long-term practicing of yoga. The results of the current study are consistent with this conclusion.

Spiritual intelligence also produced this “graded” pattern of benefits. As with interoceptive awareness and self-compassion, long-term practitioners had greater spiritual intelligence scores on the SISRI-24 compared to intermittent and non-practitioners, and the intermittent practitioners reported greater spiritual intelligence compared to non-practitioners. Few yoga studies have incorporated a quantitative measure of spirituality and compared it to psychological functioning, although there is evidence that spirituality is an important construct for yoga practitioners (Cartwright et al., 2020). The challenge for researchers is to identify the cognitive and emotional components associated with spirituality that may be influenced by yoga practice. Kelly and Moritz (2009) reported that individuals who received lectures and stories on topics of spirituality described feeling a greater sense of connection with themselves, others, and the world while also showing improved self-compassion and mood. Other researchers have suggested an important role for spiritual teachings in emotion reappraisal strategies (Menezes, 2015). It is possible that yogic spirituality helps enhance self-compassion, improve one’s mood, and develop emotional regulation strategies that assist with a practitioner’s motivation. Additional research is necessary to investigate these possibilities.

A somewhat surprising result from the current study was that the three groups did not differ on measures of depression, anxiety, or stress. This study took place during the COVID-19 pandemic and it is possible that participants exhibited elevated depression, anxiety, and stress levels. However, other studies have reported no significant differences in these, and similar constructs prior to the pandemic (Kiecolt-Glaser et al., 2010; Vollbehr et al., 2018; Papp et al., 2019). These results stand in contrast with those that showed decreased depression and anxiety scores in individuals who practice yoga (e.g., Khalsa et al., 2009; Telles et al., 2015; Snaith et al., 2018). It is unclear what accounts for these differences across studies, although a variety of measures may be factors such as difference in scales, research methodology, or differences in participant demographics. A longitudinal study that includes measures of depression, anxiety, and stress may be useful in delineating the potential protective effects of yoga practice on mental health.

The current study is one of few to investigate a mechanistic model of yoga using mediation analyses (see Boni et al., 2018). These mediation models led to several noteworthy findings. First, they demonstrate that yoga plays a role in reducing emotion dysregulation through two separate paths: a bodily (interoceptive awareness) and spiritual (spiritual intelligence) path. This notion is intriguing, because it suggests an important role for both interoceptive awareness and spiritual intelligence in improving emotional regulation through the practice of yoga for both long-term and intermittent practitioners. This is a unique finding in the literature and warrants further attention from researchers. One possible explanation is that practicing yoga improves interoceptive awareness due to its bodily focus (Emerson, 2015). Practicing yoga may enhance a sense of connection with the Self, others, and the world, contributing to a sense of spirituality (Feuerstein, 2008) and seeing life as more connected and greater than the Self alone (McGonigal, 2019). McGonigal (2019) described the importance of synchronous movement in generating a transcendent state in which people feel connected to others and as part of something greater than themselves. She explained that collective movement helps people combat loneliness, reduce pain, and enhance trust, belonging, and cooperation (McGonigal, 2019). Yoga classes that incorporate collective movement are thus likely to contribute to the aspect of spirituality related to a sense of connection with others and, possibly, the Self.

Traditional yoga philosophy supports these models. In yoga philosophy, one achieves enlightenment through penetration of the *koshas*, five sheaths that surround the Self (Feuerstein, 2008). As an individual penetrates each sheath, from the outermost to the innermost, they may eventually reach the Self and thus achieve a state of enlightenment (Feuerstein, 2008). Thus, our spiritual mediation model may be consistent with this traditional yoga philosophy if we interpret that penetrating the *koshas* enhances spirituality and could lead to improved emotional well-being. We may also interpret this traditional philosophy in another context, where penetrating each sheath is a spiritual practice in and of itself. These mediation models suggest a prominent role for traditional yoga philosophy in the interpretation of spiritual findings.

An additional noteworthy result from our mediation analyses was that yoga may operate through a cognitive path using trait mindfulness and self-compassion for both long-term and intermittent practitioners. This study suggests that trait mindfulness and self-compassion concurrently mediate the relationship between yoga experience and emotion dysregulation. In the absence of these mediators, the relationship between yoga experience and emotion dysregulation was significant, although when these mediators were added to the model, yoga experience became non-significant. This suggests that part of yoga’s mechanism for improving emotion dysregulation may be through the incorporation of mindfulness and self-compassion. Consistent with this hypothesis, Wisener and Khoury (2022) reported that trait mindfulness and self-compassion mediated the relationship between emotional regulation and eating as a coping strategy. Additionally, Per et al. (2022) demonstrated that the relationship between emotional regulation and non-suicidal self-injury was mediated by mindfulness and self-coldness (the opposite of self-compassion). When taken together with the first finding, these results highlight the potential importance of physical, mental, and spiritual influences for improving emotional regulation.

An interesting finding from our mediation analyses suggests that emotion dysregulation influenced mood. Emotion dysregulation mediated the relationship between yoga experience and depression, anxiety, or stress for both long-term and intermittent practitioners. This finding is consistent with clinical theoretical orientations that emphasize emotional regulation skills (i.e., Dialectical Behavior Therapy; Linehan, 2014) and emotional processing (i.e., Emotion-Focused Therapy; Greenberg, 2017) for improving mental health. Our finding is also consistent with other studies. Behrouian et al. (2020) found that emotional regulation training in caregivers reduced DASS-21 scores at 1-month follow-up. Patients receiving treatment intervention demonstrated reduced depression and anxiety scores, with emotional regulation as a predicting factor (Khakpoor et al., 2019). Emotional regulation was found to be a mediator between scores on the Adverse Childhood Experiences Scale (ACE) and depression (Cloitre et al., 2019). This finding appears to be robust clinically and in research, suggesting an important role for emotional regulation in influencing mood in a yoga context.

### 4.1. Limitations

The current study has several limitations that must be addressed in future research. First, the cross-sectional design limits the conclusions that can be made from the findings, specifically with regard to causality. For instance, it is possible that individuals with higher adaptive psychological functioning might be more inclined to engage in a yoga practice. There is also a possibility that certain personality features may predispose an individual to practice yoga, and the findings here are more indicative of personality traits than yoga. The use of a randomized control trial (RCT) experimental design would help address this issue in future research. Second, the solely American sample impedes cross-cultural comparisons; this limitation is important because yoga can serve different functions not only for individuals, but also in different cultures (Feuerstein, 2008). Future research should investigate whether practitioners’ intentions and culture of practice influence the magnitude of yoga’s benefits. Third, because the data were conducted in an online survey, participants did not undergo clinical screening for mental health or medical conditions. Fourth, our study did not investigate whether individuals experiencing psychological distress (or a diagnosed mental health disorder) would be more or less likely to engage in a yoga practice. This possibility could be addressed in future research by including additional questions about practitioners’ motivations for performing yoga. Finally, the division of participants into categories (i.e., non-practitioners, intermittent practitioners, and long-term practitioners) prevented us from using yoga experience as a continuous variable. That said, the use of categories *did* allow us to show the difference between long-term and intermittent practitioners in a simple manner, thus allowing researchers and the general public to easily understand the key findings from this study.

### 4.2. Conclusion

The results of this study support the continued investigation of how yoga may exert its benefits and, specifically, how the length of yoga practice (i.e., yoga experience) influences these positive effects. The findings suggest that yoga may enhance emotional regulation, trait mindfulness, interoceptive awareness, self-compassion, and spiritual intelligence for long-term practitioners. Intermittent practitioners also see some benefits, although fewer compared to long-term practitioners. Furthermore, two mediation models from this study provide novel evidence that the relationship between yoga experience and emotion dysregulation is mediated by interoceptive awareness, spiritual intelligence, trait mindfulness, and self-compassion. Three mediation models suggest that the relationship between yoga experience and depression, anxiety, and stress are mediated by emotion dysregulation. These models bring an important mechanistic consideration for yoga that may influence future research designs. Studies using pre/post methodology and long-term interventions would be helpful in identifying the specific length and intensity of practice needed to achieve these benefits. Cross-cultural study comparison would also be essential to clarify the generalizability of these models of yoga’s potential benefits.

## Data availability statement

The raw data supporting the conclusions of this article will be made available by the authors, without undue reservation.

## Ethics statement

The studies involving human participants were reviewed and approved by University of Manitoba Human Research Ethics Board. The patients/participants provided their written informed consent to participate in this study.

## Author contributions

TP and SS designed this study. TP collected the data, performed the data analyses, and wrote the first draft of the manuscript. All authors contributed to the article and approved the submitted version.

## Funding

This research was funded by a grant from the Natural Sciences and Engineering Research Council (NSERC) of Canada (grant number RGPIN-2014-03928) to SS. TP was supported by an NSERC doctoral scholarship.

## Conflict of interest

The authors declare that the research was conducted in the absence of any commercial or financial relationships that could be construed as a potential conflict of interest.

## Publisher’s note

All claims expressed in this article are solely those of the authors and do not necessarily represent those of their affiliated organizations, or those of the publisher, the editors and the reviewers. Any product that may be evaluated in this article, or claim that may be made by its manufacturer, is not guaranteed or endorsed by the publisher.
